# Effects of environmental heterogeneity on phenotypic variation of the endemic plant *Lilium pomponium* in the Maritime and Ligurian Alps

**DOI:** 10.1007/s00442-020-04806-6

**Published:** 2020-12-02

**Authors:** Carmelo Macrì, Davide Dagnino, Maria Guerrina, Frédéric Médail, Luigi Minuto, John D. Thompson, Gabriele Casazza

**Affiliations:** 1 Dipartimento di Scienze della Terra dell’Ambiente e della Vita (DISTAV), University of Genoa, Corso Europa 26, 16132 Genoa, Italy; 2 Institut Méditerranéen de Biodiversité et d’Ecologie marine et continentale (IMBE), Aix Marseille Université, Avignon Université, CNRS, IRD, Technopôle de l’Arbois-Méditerranée, BP 80, 13545 Aix-en-Provence Cedex 4, France; 3 Centre d’Ecologie Fonctionnelle et Evolutive, CNRS, 1919 route de Mende, 34293 Montpellier Cedex 5, France

**Keywords:** Climatic niche, Altitude, Distribution range, Centre–periphery hypothesis, Endemic plant

## Abstract

Geographical limits of species’ distributions are assumed to be coincident with ecological margins, although this assumption might not always be true. Indeed, harsh environments such as Alpine and Mediterranean ecosystems may favour high phenotypic variability among populations, especially those in peripheral sites. Floral traits are often found to be less variable and less affected by environmental heterogeneity than vegetative traits because variation in the former may have negative effects on fitness. For this reason, it is important to quantify variation in floral traits and plant fecundity in study range limits. The objective of the study is to examine phenotypic variation and differences in reproduction in endemic *Lilium pomponium* in the Maritime and Ligurian Alps in relation to environmental variation across its distribution range. In this species, marginal climatic populations occur both in the peripheral and central geographical locations of the distribution range; hence, geographical and ecological gradients are not concordant. Floral trait variation is related to local environmental conditions with an array of interactions among resource availability, potential pollen limitation and population size that are differentially related to floral traits. Contrary to the general expectation, all central and peripheral populations had similar, moderate seed production with each group limited by different factors acting on different stages of the life-history strategy. Our results are in line with the idea that general expectations are confirmed only when its assumptions are met and that the differences in pollination environment along an environmental gradient may not be the main determinant of the distribution limit.

**Electronic supplementary material:**

The online version of this article (10.1007/s00442-020-04806-6) contains supplementary material, which is available to authorized users.

## Introduction

There is much interest in ecology and evolution in the occurrence of trait variation from the centre towards the geographical periphery of species’ distributions (Sagarin and Gaines 2002; Eckert et al. 2008; Pironon et al. 2015). In particular, as the centre–periphery hypothesis (CPH) predicts, geographically isolated peripheral populations are expected to be divergent from central populations and to be smaller, less abundant and more isolated from each other than central populations, features that are likely to significantly affect levels of both neutral and adaptive genetic diversity when compared to central populations (Pironon et al. 2017). In plants, a decline in habitat quality towards the periphery of the range is expected to cause either a decline in population size and density resulting in reduced pollinator service (Stone and Jenkins 2008; Moeller et al. 2012) or inadequate pollination driving the decline of population size (Hegland and Totland 2005; Moeller et al. 2012) and, whatever the case, little potential for further range expansion. These responses assume that environmentally marginal populations occur at the geographical periphery of species range with inadequate pollination at the range margins. The loss of stigma-height polymorphism in peripheral populations of different *Narcissus* species in the Mediterranean region fit this schema (Barrett et al. 2004; Papuga et al. 2015).

However, the assumption of concordance between geographical periphery and environmental marginality has received increasing criticism (Soulé 1973; Pironon et al. 2017). First, peripheral populations may occur in conditions similar to those in the centre of the range (Piñeiro et al. 2007; Kropf et al. 2008). Second, environmental factors may impose ecologically marginal conditions in any part of the species’ range (Soulé 1973—hereafter “marginal populations”). Third, geographically peripheral populations may not occur in marginal conditions but simply in different ecological conditions (Papuga et al. 2018). In particular, in Alpine and Mediterranean ecosystems, environmental factors change over very short distances because of the high topographic complexity (e.g. Körner 2003; Thompson 2001; Doxa and Prastacos 2020), leading to differences in abiotic and biotic resources not necessarily associated with different parts of the range. Such highly heterogeneous and mosaic environments may favour high phenotypic variability (Graae et al. 2018). For all these reasons, geographically peripheral populations are of fundamental importance to studies of species’ range limits (Lesica and Allendorf 1995; Hampe and Petit 2005).

In general, floral traits are found to be less variable and less affected by environmental heterogeneity than vegetative traits because variation in floral morphology may have negative effects on fitness (Berg 1960; Frazee and Marquis 1994). Nevertheless, population variation has been detected in stigma–anther separation (Griffin and Willi, 2014; Papuga et al. 2015), floral display (Dai et al. 2017; Lambrecht et al. 2017), and pollen–ovule ratio (Guo et al. 2010; Dai et al. 2017). This variation in floral traits is usually related to variation in the pollination environment (Aigner 2004) and some studies have detected increased pollen limitation at the distributional edges (Moeller et al 2012), although others do not record any increase in pollen limitation, presumably due to increase in resource constraints or self-pollination (Totland 2001; Hargreaves et al 2015). Assessing the relationship between environmental variation and traits related to pollination environment may increase our understanding of factors shaping distribution limits and species persistence. However, relatively few studies have been conducted to understand the relationship between floral trait variation and environmental variation across the range (but see Gamble et al 2018; Seguí et al 2018).

**Fig. 1:**
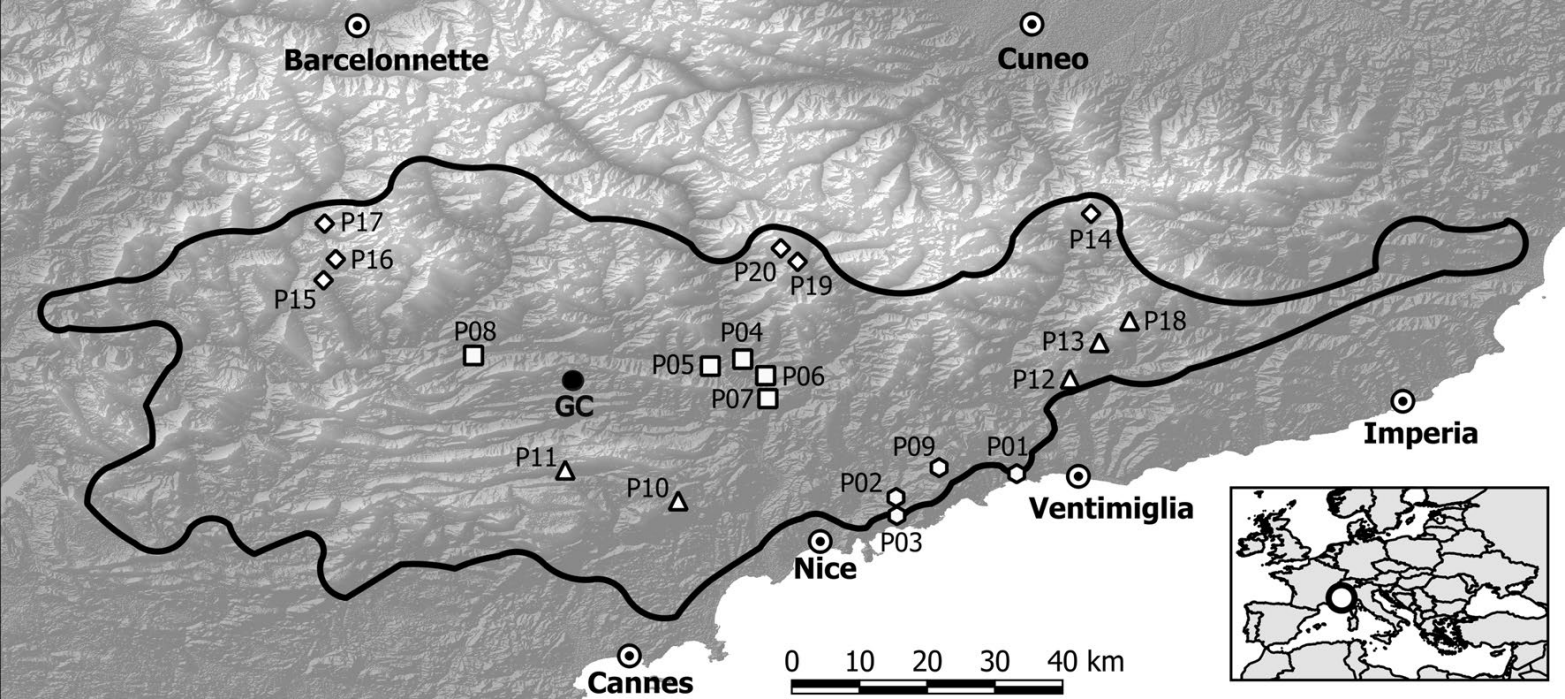
Map showing the geographic location of the 20 populations of *Lilium pomponium* used in this study. Symbols indicate the four climatic groups: coastal marginal (hexane ring), central populations (unfilled up-pointing triangle); inland marginal (unfilled rectangle), and subalpine marginal (unfilled Lozenge). Filled circle = geographic centre (GC). Populations’ codes are those reported in Table 1

*Lilium pomponium* L. is a self-incompatible perennial geophyte endemic to the Maritime and Ligurian Alps (Fig. 1) that grows on calcareous outcrops from 100 to 2000 m altitude, from a typical Mediterranean climate to a cool-summer continental type climate in subalpine habitats (Casazza et al 2018). This species thus provides an excellent model for studying traits related to pollination across an environmental gradient. The objective of the present study is to examine whether phenotypic and reproductive traits are related to environmental and geographical gradient across the distribution range of *L. pomponium* L. Because of the rough topography of the study area, we explicitly took into account climatic conditions rather than using altitude as their proxy in assessing environmental marginality. Specifically, the goals of this study were (1) to assess whether geographically peripheral populations are also environmentally marginal; (2) to assess whether these environmentally marginal populations differ phenotypically from central populations in floral traits, and whether they show differences in reproductive output and mating system.

## Materials and methods

### Study species

*Lilium pomponium* L. (Liliaceae) has hermaphrodite flowers although male flowers can occur (ca. 10%, personal observation). Anthesis usually lasts from May to July and capsules develop from late July to September, according to local climatic conditions. Reproductive output is low, flowers are self-incompatible (Casazza et al. 2018) and show ‘*approach herkogamy*’, that is, the stigmas are above the anther levels (Fryxell 1957; Webb and Lloyd 1986). These traits limit self-pollination and promote outcrossing (Webb and Lloyd 1986) and reduce sexual interference (Barrett 2002). Seeds are very thin with a winged margin for wind dispersal (pterometeorochory) that enables herbs to reach roughly 15 m (Vittoz and Engler 2007).

### Study occurrences and climatic data

The distributional range of *L. pomponium* extends from the Neva Valley in northwest Italy to the Verdon Valley in southwest France (Fig. 1). Species occurrences were obtained from field surveys (performed by the authors) and from regional databases: SILENE (Conservatoire botanique national méditerranéen de Porquerolles; http://flore.silene.eu/) and LiBiOss (Regione Liguria; http://www.cartografiarl.regione.liguria.it/Biodiv/Biodiv.aspx). Occurrences were spatially filtered and those closer than 1 km to each other were removed, resulting in a final data set of 809 occurrences. To study climatic conditions of populations, we downloaded nineteen bioclimatic variables representative of the period 1979–2013 from the CHELSA climate database website (http://chelsa-climate.org/) at 30-s (c. 1 km) spatial resolution (Karger et al. 2017).

### Definition of climatically central and marginal populations

To distinguish ecologically marginal and central populations we first characterized the climatic niche of *L. pomponium* carrying out a principal component analysis (PCA) of the bioclimatic variables using the ‘ade4’ package implemented in R (R Core Team 2018). We considered “central” and “marginal” the populations falling into and outside the 70% of confidence ellipse, respectively. Then we grouped the marginal populations in different climate groups according to the PCA quadrants where they fall. Moreover, we tested differences among groups of populations because of mean annual temperature and annual precipitation using the non-parametric Kruskal–Wallis test. Kernel density plots were used to visualize the distribution of each variable.

### Correlation between geographical and climatic distance

To test whether populations that are geographically peripheral are also ecologically different or marginal, we calculated the Euclidean distance from each population to the centroid of climatic space in the PCA and the Euclidean distance from each population to the centre of the distributional range. We calculated the correlation between the two distances using Spearman rank correlation coefficient.

### Phenotypic variation

**Fig. 2:**
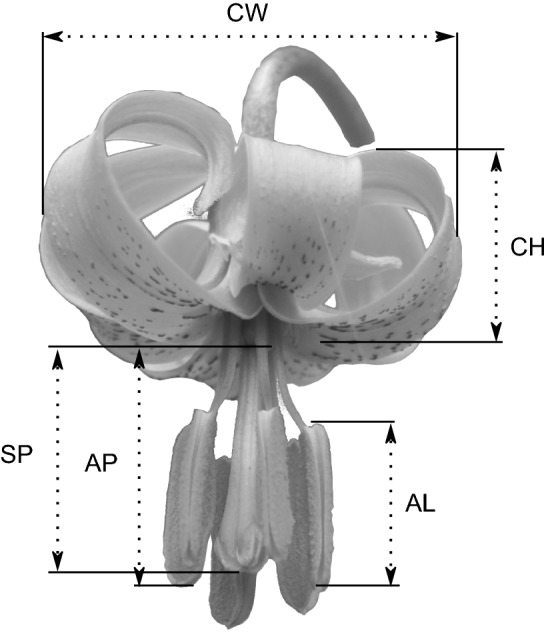
Floral trait measurements. *CW* corolla width, *CH* corolla height, *SP* stigma position, *AP* anther position, *AL* anther length. Measurements of SP and AP were taken from the top of the ovary

To assess whether different groups of populations differ in floral traits, we measured 562 flowers from 414 randomly chosen plants ranging from 8 to 34 per populations (according to population size) during the years 2017–2018 (250 and 312 flowers in 2017 and 2018, respectively). The number of flowers analysed per population ranged from 13 to 80 (Table 1 and Online Resource 1) and roughly 75% of measures were from single flowers on different plants. We analysed two traits involved in pollinator attraction: the number of flowers per scape and the corolla surface. In particular, corolla surface was calculated as the surface of an oblate spheroid (Fig. 2), measuring height and width of the corolla (the latter measured three times, one for each pair of tepals—CH and CW, respectively, in Fig. 2). We also analysed a trait involved in flower–pollinator interaction: the spatial separation of pollen presentation and pollen receipt; in particular, we measured the distance between the top of the ovary and the tip of the stigma (stigma position: AP in Fig. 2), the distance between the top of the ovary and the tip of the six stamens (anther position: AP in Fig. 2), and the length of the six anthers (AL in Fig. 2). We classified flowers in three categories: (1) flowers showing approach herkogamy, stigma above the anther; (2) flowers showing reverse herkogamy, stigma below the anther; and (3) flowers without herkogamy, stigma among the anthers. Floral measurements were obtained in field with a digital caliper (error =  ± 0.01 mm).

**Table 1:** Locations and populations characteristics of the 20 populations of *Lilium pomponium* used in this study

Pop	Group	Country	Lat	Long	Alt	Pop size	No. measured flowers
P01	CM	Baisse Saint-Paul, Castellar (IT)	43.793	7.526	434	~ 100	25
P02	CM	Plateau Tercier, Sainte-Thècle (FR)	43.755	7.369	554	~ 250	33
P03	CM	Fort de la Revère (FR)	43.737	7.367	661	~ 40	16
P04	IM	Les Pras, La Tour (FR)	43.944	7.163	243	~ 50	10
P05	IM	Ciamp du Var, Maisson (FR)	43.935	7.120	267	~ 30	10
P06	IM	Route de la Tinée, Tournefort (FR)	43.919	7.187	230	~ 200	27
P07	IM	Route de Grenoble, Utelle (FR)	43.905	7.196	203	~ 100	20
P08	IM	Entrevaux (FR)	43.949	6.805	586	~ 20	17
P09	CM	Col de la Madone de Gorbio, Peille (FR)	43.801	7.423	915	~ 200	30
P10	CC	Col De Vence, Vence (FR)	43.757	7.0775	955	~ 200	31
P11	CC	Greolieres (FR)	43.797	6.9276	1014	~ 150	25
P12	CC	Mt. Comune, Pigna (IT)	43.918	7.5975	1123	~ 150	30
P13	CC	Mt. Lega, Pigna (IT)	43.966	7.6363	1326	~ 200	23
P14	SM	Castel Tournou, Tenda (FR)	44.125	7.6254	1334	~ 30	14
P15	SM	Méailles (FR)	44.037	6.6068	1313	~ 250	35
P16	SM	Peyresq (FR)	44.964	6.6231	1466	~ 70	20
P17	SM	Ondres (FR)	44.112	6.6082	1565	~ 200	31
P18	CC	Mt. Grai, Pigna (IT)	43.995	7.6772	1759	~ 500	40
P19	SM	L'adrechas, La Colmiene (FR)	44.075	7.2255	1646	~ 400	80
P20	SM	La Colmiene (FR)	44.078	7.2174	1789	~ 200	43

Population size was estimated by counting the number of flowering individuals. See Fig. 1 for a map of the study locations

Group refers to climatic groups: *CM*, coastal marginal; *IM*, inland marginal; *CC*, central and *SM*, subalpine marginal

### Pollen limitation and reproductive performances

To test whether the self-fertilization rate was different between groups of populations, we bagged 183 flower buds from 139 plants (ranging from 10 to 20) in 12 populations using non-woven fabric bags. Furthermore, to test whether the degree of pollen limitation leading to reduced seed production was different between central and marginals populations, we assigned a total of 168 flowers from 143 plants (ranging from 3 to 28) in 17 populations to supplemental hand pollination (Ps) and 344 flowers from 279 plants (ranging from 4 to 42) in 17 populations to open pollination as control (Online Resource 1).

For each population, we quantified seed number on naturally pollinated flowers (Po) and supplementary-pollinated flowers (Ps) and thus obtained a value for pollen limitation (PL) using the formula of Baskin and Baskin (2017): PL = (Ps − Po)/Pmax [Ps or Po]. Values of PL range from 1 to − 1. Positive values indicate a lower seed set in natural than in pollen-supplemented flowers, negative values indicate a higher seed set in natural than in pollen-supplemented flowers. Because the number of pollen donors may affect the reproductive success (Schemske and Pautler 1984), we collected pollen from at least three donors.

To estimate Ps (seed set in pollen-supplemented flowers) and Po (seed set in open-pollinated flower), mature capsules were collected before dehiscence, preventing seed dispersion. Seeds were counted under a Leica M205 C stereomicroscope. We calculated seed set as filled seeds/total number of ovules (filled seeds + aborted seeds + unfertilized ovules).

### Statistical analyses

To test whether the groups of populations differed significantly in number of flowers per scape, flower size, and pollen and ovules production, we applied the non-parametric Tukey–Kramer–Nemenyi post hoc test using the R ‘PCMMRplus’ package (Pohlert 2014), implemented in R (R Core Team 2018). To test whether the groups of populations differed significantly in the percentage of flowers belonging to different groups of populations, we used chi-squared or exact Fisher test when the expected frequencies was less than five in some cells.

Because seed production follows a binomial distribution, lacking the property of linearity and additivity, the effects of marginality of populations on plant fitness were analysed by fitting factorial generalized linear mixed models (GLMMS, logit link function, binomial distribution) to the seed set data with group of populations as fixed predictors, and populations as random factor. Statistical analyses were performed using the ‘lme4’ package (Bates et al. 2015) implemented in R (R Core Team 2018). Post hoc tests were conducted to evaluate pair-wise differences in measured traits between treatments using the ‘glht’ function in the ‘multicomp’ package (Hothorn et al. 2008) implemented in R (R Core Team 2018). Moreover, to test the relationship between elevation and flower size, number of flowers per scape, seed set and degree of pollen limitation, we calculated Spearman rank correlation coefficients.

## Results

### Central and marginal populations

**Fig. 3:**
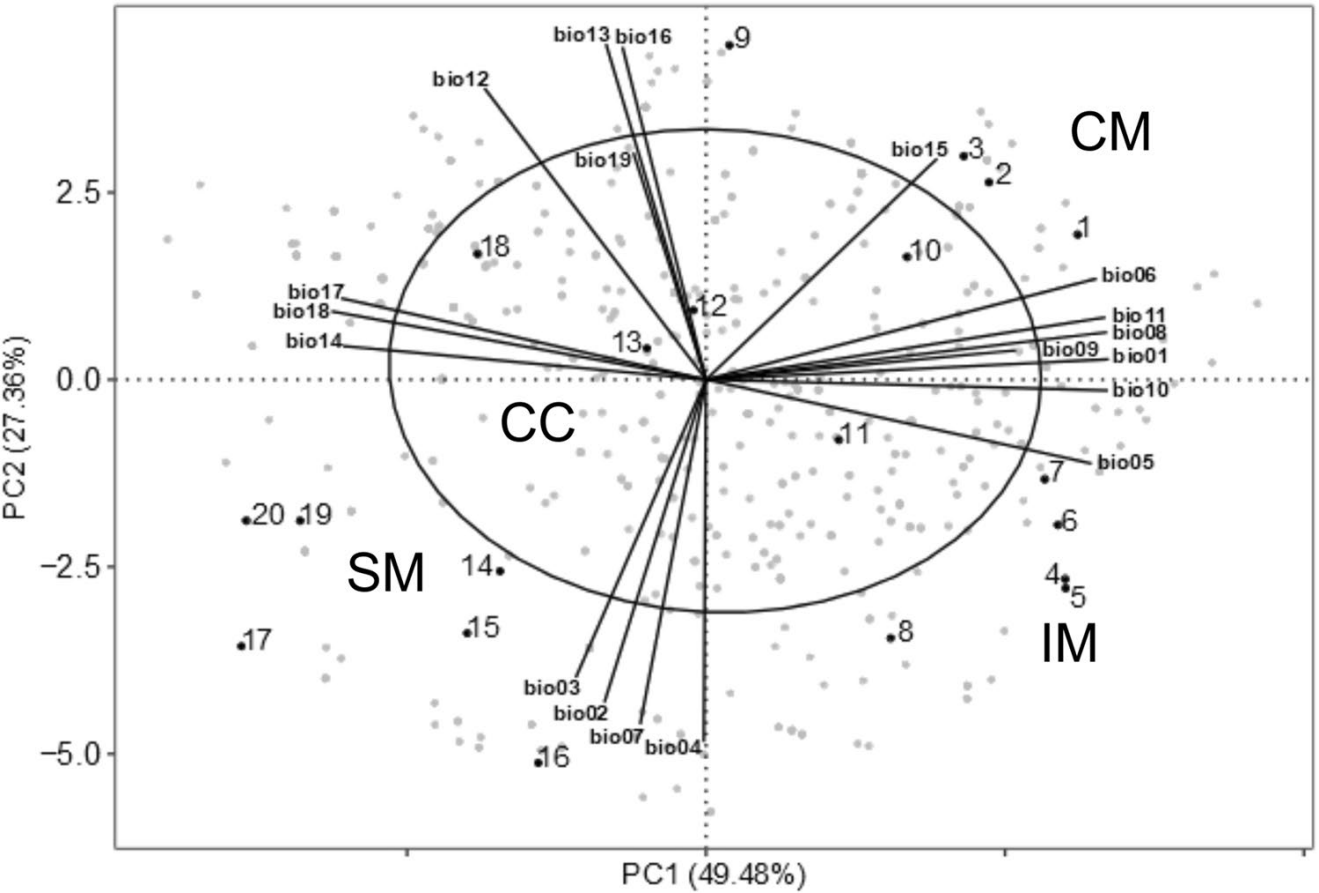
Principal component analysis of climate data for all known population locations of *Lilium pomponium* populations (grey dots). Ellipses include 70% of each class variance. Central populations are inside the ellipse while the marginal populations are outside. Numbers indicate the sampled populations (see Table 1). P01, P02, P03, P09: coastal marginal (CM); P04, P05, P06, P07, P08: inland marginal (IM); P10, P11, P12, P13, P18: central (CC); P14, P15, P16, P17, P19, P20: subalpine marginal (SM). Populations’ codes are those reported in Table 1. The nineteen bioclimatic variables (BIO01-BIO19) were downloaded from the CHELSA climate database website [https://chelsa-climate.org/]

**Fig. 4:**
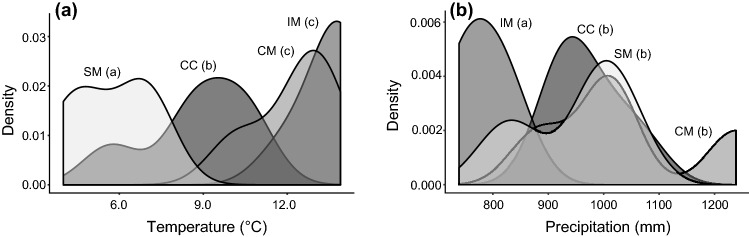
Kernel density plots of average annual **a** temperature and **b** precipitation experienced by populations of *Lilium pomponium* in the four different sampled climatic conditions: *CM* coastal marginal populations, *IM* inland marginal populations, *CC* central population, and *SM* subalpine marginal populations. Different letters indicate statistical differences (i.e., *P* values ≤ 0.05). *P* values were calculated using the non-parametric Kruskal–Wallis test

The first two axes of the PCA explain 76.84% of the variation of the whole data set. The ellipses drawn include 70% of the data of the group. This allowed us to recognize five central populations (hereafter CC) and 15 marginal populations (Fig. 3). The marginal populations were further subdivided into three different groups growing under different climatic conditions (Fig. 4). The first group, hereafter called coastal marginal (CM) included four populations growing under warm and moist conditions, probably due to due to the vicinity to the sea (Figs. 3, 4). The second group, hereafter called inland marginal (IM) included five populations growing under warmest and driest conditions (Figs. 3, 4). The third group, hereafter called subalpine marginal (SM) included six populations of the subalpine belt growing under cold and wet conditions (Figs. 3, 4). Distance from the geographical centre and distance from the climatic centre were negatively correlated (*ρ* = − 0.17, *p* value = 0.48).

### Phenotypic variation

**Fig. 5:**
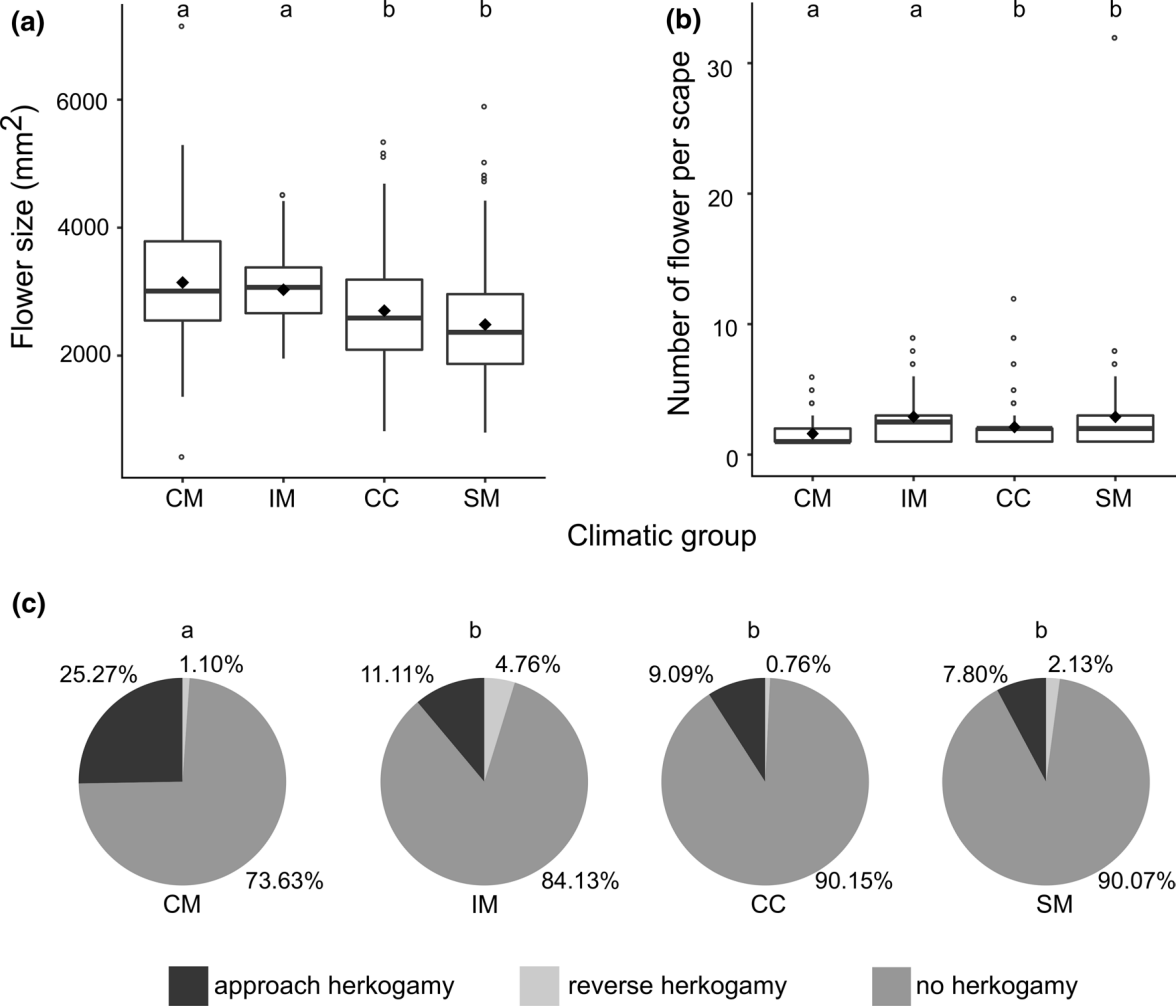
Boxplot of corolla surface. **a** Flower size, **b** number of flowers per scape and **c** extent of herkogamy in populations of *Lilium pomponium* in four sampled climatic groups: coastal marginal populations (CM), inland populations (IM), central populations (CC), and subalpine marginal populations (SM). **c** Percentage of flowers with a separation between stigma and anthers. Different letters indicate statistical differences (i.e., *P* values ≤ 0.05). *P* values were calculated using non-parametric Tukey–Kramer–Nemenyi post hoc test to assess difference in flower size and number of flowers per scape and using chi-squared or exact Fisher test to assess difference in herkogamy

Flowers were significantly larger at low elevation in CM and IM (Fig. 5a); mean flower size decreased from 3.14 mm^2^ (CM), 3.03 (IM), 2.70 (CC) to 2.49 (SM) mm^2^. The number of flowers per scape was significantly lower in CM and CC (Fig. 5b). In particular, CM and CC plants bore 1.61 (sd = 1.07) and 2.12 (sd = 1.86) flowers, while SM and IM plants bore 2.89 (sd = 4.64) and 2.90 (sd = 2.06) flowers. In the majority of flowers (86%), the stigma was placed at the level of anthers (i.e., no herkogamy); 12% of flowers showed approach herkogamy and 2% of flowers showed reverse herkogamy. A significant difference in percentage of flowers with a separation between stigma and anthers was detected only between CM and the other groups (Fig. 5c). In particular, CM showed at the same time (Fig. 5c) the highest percentage of flowers with approach herkogamy (25.27%) and thus the lowest percentage of flowers without herkogamy (73.63%). Hence, flower size is significantly, positively correlated with elevation and approach herkogamy is particularly frequent in CM, where three out of four populations had a high proportion of flowers with marked approach herkogamy (Online Resource 2 Fig. S1a, c). In contrast, no correlation between the number of flowers per scape and elevation was detected (Online Resource 2 Fig. S1b).

### Pollen limitation and reproductive performances

**Fig. 6:**
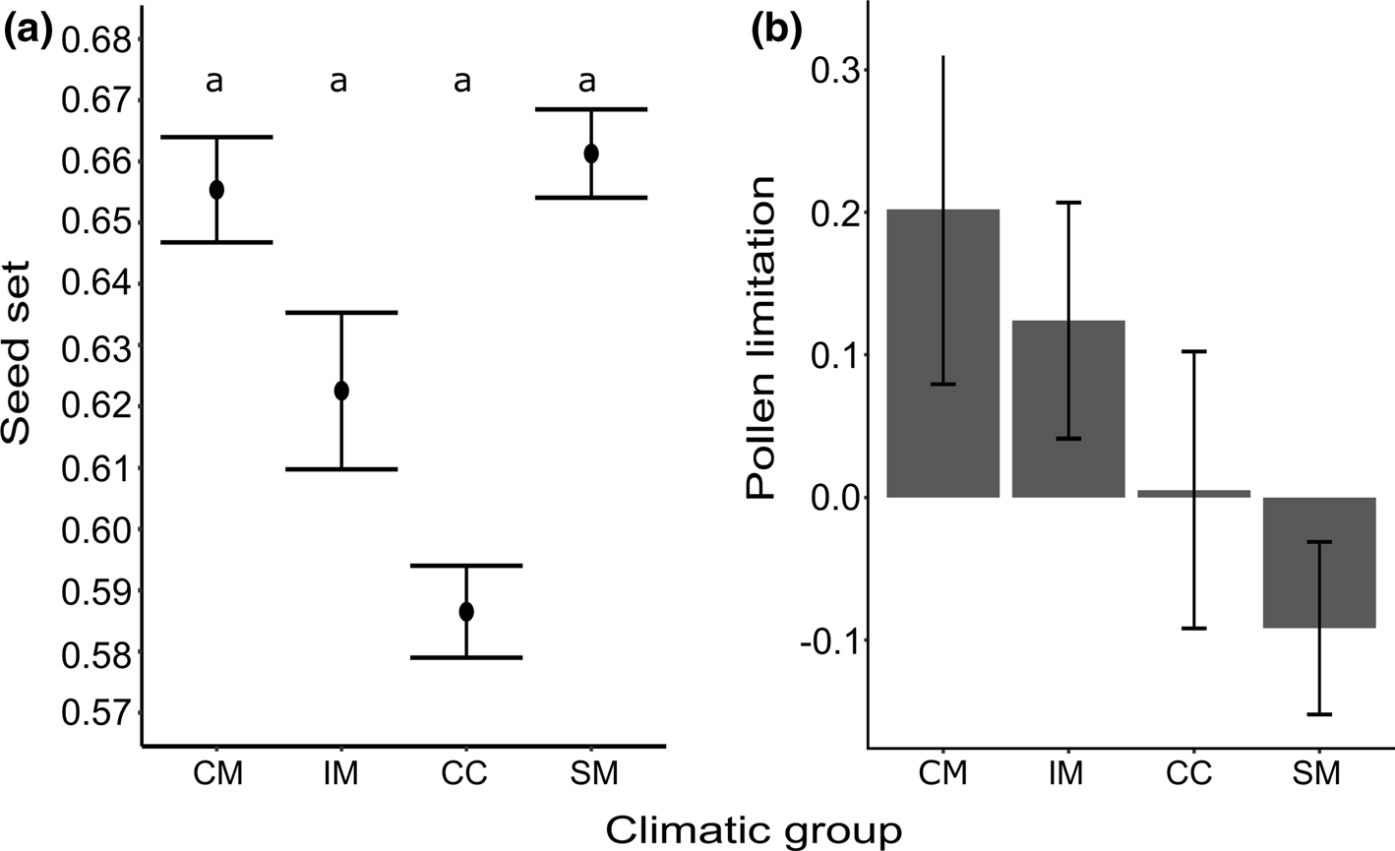
Comparisons of **a** seed set (mean ± 95% confidence interval) and **b** pollen limitation (mean ± standard error) in populations of *Lilium pomponium* in four sampled climatic groups: coastal marginal populations (CM), inland populations (IM), central populations (CC), and subalpine marginal populations (SM). Seed set was calculated as filled seeds/filled seeds + aborted seeds + unfertilized ovules and pollen limitation was calculated as: (Ps − Po)/Pmax [Ps or Po] where Po = seed number in naturally pollinated flowers and Ps seed number in supplementary-pollinated. Different letters indicate statistical differences (i.e., *P* values ≤ 0.05). *P* values were calculated using a general linear hypothesis tests

No significant differences were detected in seed set among groups of populations. Seed set was pollen-limited mainly in CM (PL = 0.20) and in IM (PL = 0.12). In CC the mean value of PL was close to zero (PL = 0.005), suggesting no pollen limitation. Differently, the mean PL value of SM (Fig. 6) was weakly negative (PL = − 0.09). In the self-pollination treatment, only one out of the 93 flowers produced a fruit. Hence, seed set was not significantly correlated with altitude while the level of pollen limitation was significantly negatively correlated with altitude (Online Resource 2 Fig. S2a, b).

## Discussion

### Congruence between geographical and environmental marginality

According to the central–peripheral hypothesis (CPH), species are predicted to have higher performance in the centre of their distributional range where habitat conditions are expected to be more favourable and stable than in peripheral or marginal populations (Hengeveld and Haeck 1982; Brown 1984). In fact, the harsh environmental conditions at the periphery may result in a decline in pollinator service, such that populations at geographical and environmental edges are strongly pollen limited (Moeller et al. 2012), or in a decline in population size that causes less pollinator attraction (Hargreaves and Eckert 2014). In both cases, a reduction in seed set and an increase in pollen limitation occur due to a change in the pollination service in peripheral populations.

In *L. pomponium*, populations in marginal climatic groups CM and SM occur, respectively, at the southern and northern geographical extremes of the distributional range, while the marginal environmental group IM occurs closer to the centre of the distributional range than populations in the central group CC (squares and triangles in Fig. 1). In particular, environmentally marginal populations grow in warm and dry conditions (Fig. 4) and are located both at southern (i.e., CM group) and lower altitudinal (i.e., IM group) limits, while marginal populations growing in cold and wet conditions (i.e., SM group in Fig. 4) are located at highest altitude in the northern limit of the distributional range (Table 1 and Fig. 1). This result is in line with the lack of correlation between geographical and climatic distances (i.e. *p* value 0.19 and tau − 0.22), suggesting that populations near the geographical centre are not necessarily near the ecological centre of the species niche and vice versa. These results support the idea that geographical and environmental gradients are not necessarily concordant (Ribeiro and Fernandes 2000; Herlihy and Eckert 2005; Herrera and Bagaza 2008; Villellas et al. 2012; Pironon et al. 2015; Dallas et al. 2017) and that factors such as topography and environmental heterogeneity may impose marginal ecological conditions near the geographical centre (Soulé 1973; Doxa and Prastacos 2020).

### Relationship between phenotypic and environmental marginality

Changes in the pollination environment due to changes in environmental conditions are expected to drive differentiation of floral traits between marginal and central populations. In particular, marginal populations are expected to diverge from central populations because they have smaller and fewer flowers, reduced stigma–anther separation, and high self-fertilization rate because of a reduction in pollinator visitation and less outcross pollination (Herlihy and Eckert 2005; Mimura and Aitken 2007). This pattern is not consistent among population groups in *L. pomponium*; the two environmentally marginal groups growing at the warm margin (i.e., the geographical peripheral CM and the geographical central IM) have similar traits and are rather different from central group. In particular, they diverge from CC by their pollinator-limited seed set (Fig. 6b and Online Resource 2 Fig. S2b) and significantly wider flowers (Fig. 5a and Online Resource 2 Fig. S1a). Moreover, CM populations have a higher percentage of flowers with approach herkogamy (Fig. 5c and Online Resource 2 Fig. S1c), i.e. a protruding stigma that reduces self-pollen deposition (Webb and Lloyd 1986) and IM has a high number of flowers per scape (Fig. 5b and Online Resource 2 Fig. S1b).

These results are congruent with the expectation that pollen limitation may select for enhanced attraction and favour large flowers that enhance visibility and, therefore, pollinator attraction (Thompson 2001; Arista and Ortiz 2007; Barrio and Teixido 2014) and favour the reliability of visits (Haig and Westoby 1988; Totland 2001; Teixido and Aizen 2019). Indeed, pollen-limited populations of *L. pomponium* have large flowers that may be favoured because they are more attractive to Lepidoptera (Thompson 2001), the main pollinators of *L. pomponium* (Casazza et al. 2018). Moreover, in the strongest pollen-limited group CM (PL = 0.20), the high percentage of flowers with a protruding stigma may contribute to limit self-pollen deposition that can cause self-interference that may reduce female fitness (Webb and Lloyd 1986; Li et al. 2013). In IM, the flowers are large and numerous (Fig. 5b and Online Resource 2 Fig. S1b), contrary to the general expectation of a trade-off between flower size and number because of energetic constraints (Sargent et al. 2007). Nevertheless, in this group, the high number of flowers per scape may be related to the small size of populations (Table 1). In fact, the high number of flowers may increase the frequency of within-plant pollinator movements favouring pollination of flowers by pollen from other flowers on the same plant (Mustajärvi et al. 2001; Iwaizumi and Sakai, 2004). In small-sized and pollen-limited populations (i.e., IM) of non-autogamous species, like *L. pomponium* (Casazza et al. 2018), this strategy may allow the production of a regular but low number of seeds (Roberts et al. 2014), even if it reduces outcrossing (Lloyd 1992; Harder and Barrett 1995).

In contrast, populations in the group growing in cold conditions (i.e., SM) are similar to those in the central group in that they show no evidence of pollinator limitation (Fig. 6b and Online Resource 2 Fig. Sb) and have small flowers, even though in SM they are numerous per scape (Fig. 5b Online Resource 2 Fig. S1b), according to the trade-off between flowers size and number. In SM the large number of flowers per scape might be a bet-hedging strategy to assure reproduction in unpredictable environments (Koops et al. 2003). In fact, despite their cost, the late-blooming flowers may act as a reserve when the earlier blooming ones are lost early in the season (Brown 1984), because of late spring frosts. Moreover, the seed set of SM is similar to that detected in pollen limited groups and fits with the observation that in cold environments low temperatures alone, or because they reduce pollinator activity, limit seed production (Totland 2001). In species with green photosynthetic fruits during seed maturation like *L. pomponium*, low temperatures may be particularly effective in constraining photosynthetic activity and thereby the amounts of resource allocated to seed development (Totland 2001). This result is in line with the idea that seed set may be limited by pollen receipt and/or resource availability (Haig and Westoby 1988). In fact, groups growing in warm conditions are limited more by pollen than resources and have large flowers. In contrast, the group growing in cold conditions is limited by resources and not by pollen and has small flowers.

Contrary to the general expectation of reduction of pollination in the environmentally marginal populations, in our study all groups have a quite similar and moderate seed set (ranging from 0.586 to 0.655, Fig. 6a and Online Resource 2 Fig. S2a). While the moderate seed set in marginal groups may be explained by pollen limitations or by an effect of low temperatures, the moderate seed set in the large populations of the central group (Table 1) may be explained by other non-mutually exclusive factors such as seed predation and herbivory (Garwood and Horvitz 1985; Knight et al. 2006; Straka and Starzomski 2015). For example, in groups growing in wetter and cool conditions like CC and in part also SM, seeds may be more prone to damages by the lily beetle (*Lilioceris lilii* Scopoli, 1763) that prefers shaded, cool and moist areas in mountain habitats (Majka and LeSage 2008). Moreover, the generally moderate seed set may be conditioned by the life-history strategy of *L. pomponium*; like other long-lived herbaceous perennials, the species may have a strategy of annually limited but inter-annually constant seed production, in which sub-maximal seed production is a part of a size-dependent strategy that maximises life-time seed production, without compromising adult survival (García and Zamora 2003; Andrieu et al. 2007).

Our results suggest that in *L. pomponium* not all environmental marginal groups differ from the central one, because local environmental condition resulting in an array of interaction among resource availability, biotic interactions and population size may differentially affect seed set and phenotypic variation in floral traits. The phenotypic variability in floral traits among populations in different environments may be due to plasticity—the ability of one genotype to alter its phenotype in response to environmental conditions—and/or genetic variation—an increase in the frequency of genotypes that have traits enhancing fitness. Our data were recorded in natural populations; hence, greenhouse experiment will be necessary to discriminate between these two non-exclusive possibilities.

## Conclusion

The lack of separation between geographically peripheral and central groups in traits related to pollination environment and the occurrence of an environmental marginal group near the geographical centre are in line with the idea that CHP predictions are confirmed only when its assumptions are met (Lira-Noriega and Manthey 2014; Kennedy et al 2020). Our results suggest that variability in local conditions drives variation in floral traits and probably in the pollination environment. However, the differences in pollination environment related to marginal environments along a gradient is not a main determinant of the distribution limit of *L. pomponium*, as suggested by the similar seed set values recorded throughout the distributional range. In species with predominantly localised dispersal such as *L. pomponium* fine-scale landscape heterogeneity at the geographical periphery may influence population survival due to an inability to persist below a threshold of density (Keitt et al. 2001) irrespective of pollination success.

## Electronic supplementary material

Below is the link to the electronic supplementary material.

Supplementary file1 (DOCX 610 KB)

Supplementary file2 (DOCX 18 KB)

## Data Availability

The datasets generated during the current study are available in the ZENODO digital repository, https://doi.org/10.5281/zenodo.3757739 and https://doi.org/10.5281/zenodo.3766851.
