# Supplementary material for: Effects of environmental heterogeneity on phenotypic variation of the endemic plant Lilium pomponium in the Maritime and Ligurian Alps
Source: Oecologia. 2020 Dec 2;195(1):93–103. doi: 10.1007/s00442-020-04806-6 (PMC7882563; doi:10.1007/s00442-020-04806-6)
Supplement: Supplementary file 1 — Supplementary file1 (DOCX 610 KB) [file 442_2020_4806_MOESM1_ESM.docx]

**ONLINE RESOURCES**

**Article**: Effects of environmental heterogeneity on phenotypic variation of the endemic plant Lilium pomponium in the Maritime and Ligurian Alps

**Journal**: Oecologia

**Authors:** Carmelo Macrì, Davide Dagnino, Maria Guerrina, Frédéric Médail, Luigi Minuto, John D. Thompson, Gabriele Casazza

**Corresponding author:** Luigi Minuto, Department of DISTAV, University of Genoa, Corso Europa 26, 16132, Genoa, Italy luigi.minuto@unige.it


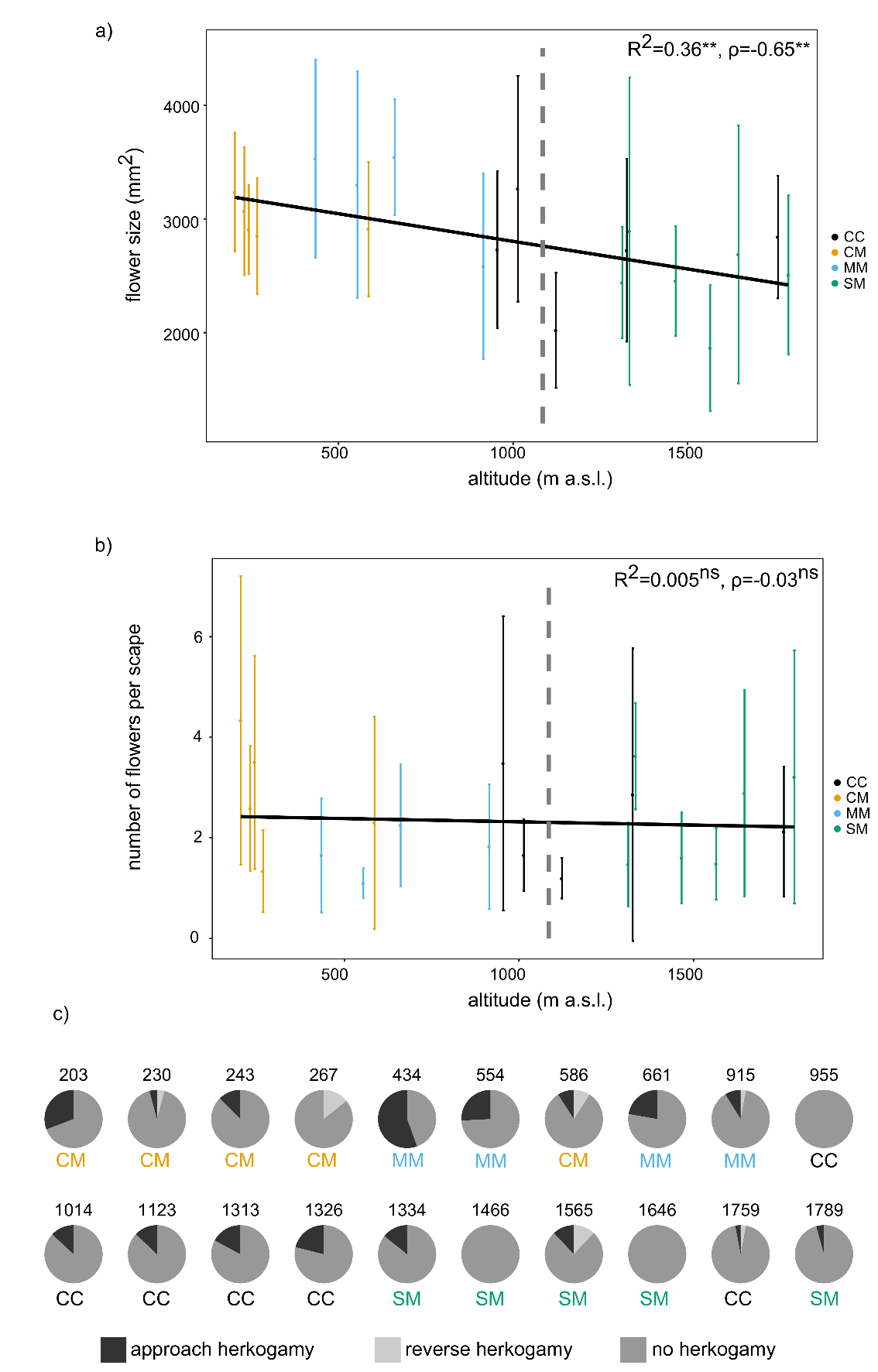


Figure S1: Bar plot showing the relationship between average corolla surface and altitude of populations. b: Bar plot showing the relationship between average number of flowers per scape and altitude of population. c: percentage of flowers with a separation between stigma and anthers in populations sorted by altitude. Error bars indicate standard deviations; black lines are regression lines. ρ indicates Kendell-tau correlation coefficients. MM = Mediterranean marginal populations; CM = central marginal populations; CC = central populations; SM = subalpine marginal populations. ** *P*-values ≤ 0.01, ^ns^ *P*-values > 0.05.


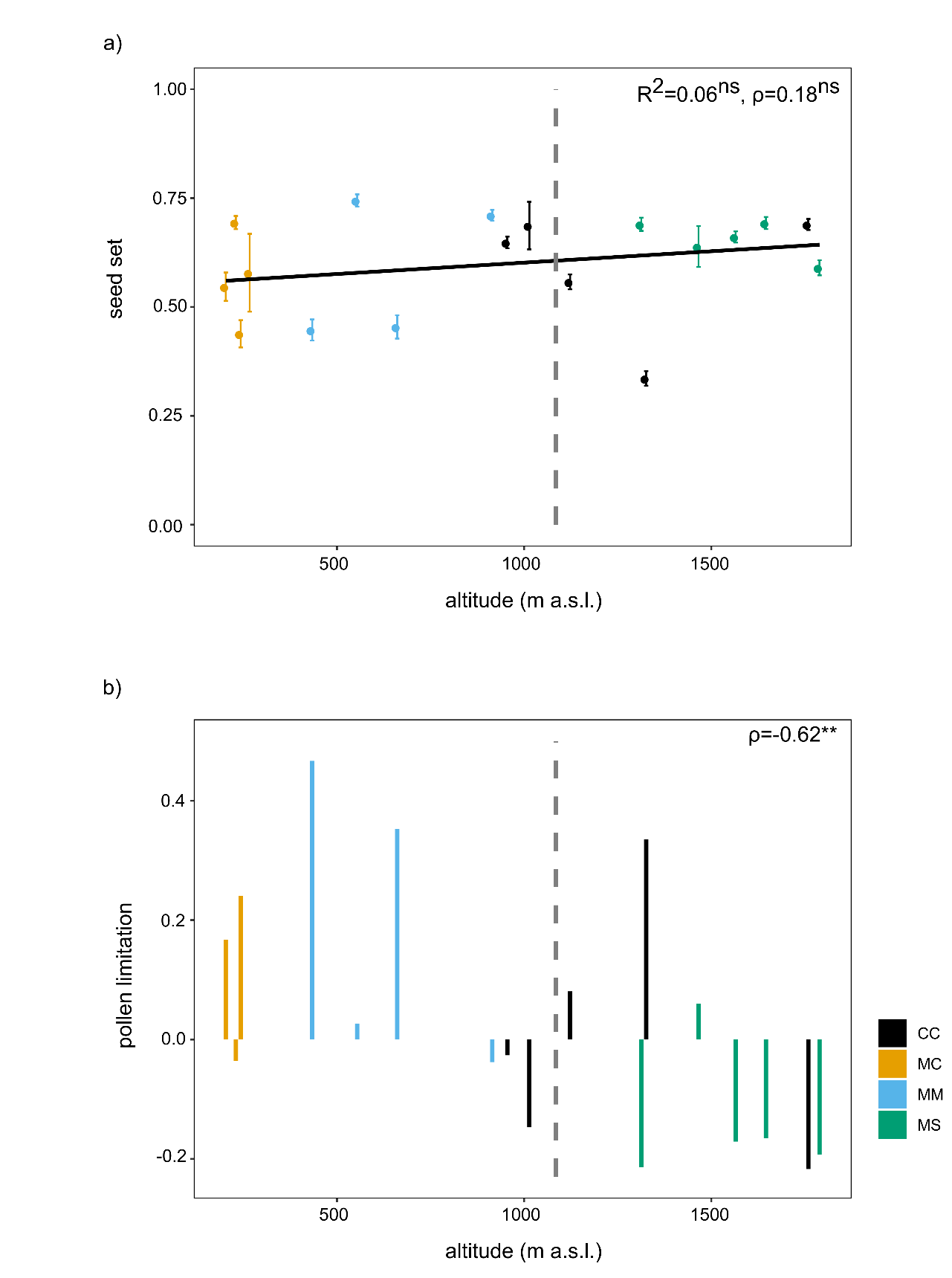


Figure S2: Bar plot showing the relationship between mean seed set values and altitude of populations. b: Bar plot showing the relationship degree of pollen limitation and altitude of population. Error bars indicate standard deviations; black lines are regression lines. ρ indicates Kendell-tau correlation coefficients. MM = Mediterranean marginal populations; CM = central marginal populations; CC = central populations; SM = subalpine marginal populations. ** *P*-values ≤ 0.01, ^ns^ *P*-values > 0.05.
