# Supplementary material for: Effects of environmental heterogeneity on phenotypic variation of the endemic plant Lilium pomponium in the Maritime and Ligurian Alps
Source: Oecologia. 2020 Dec 2;195(1):93–103. doi: 10.1007/s00442-020-04806-6 (PMC7882563; doi:10.1007/s00442-020-04806-6)
Supplement: Supplementary file 2 — Supplementary file2 (DOCX 18 KB) [file 442_2020_4806_MOESM2_ESM.docx]

**ONLINE RESOURCES**

**Article**: Effects of environmental heterogeneity on phenotypic variation of the endemic plant Lilium pomponium in the Maritime and Ligurian Alps

**Journal**: Oecologia

**Authors:** Carmelo Macrì, Davide Dagnino, Maria Guerrina, Frédéric Médail, Luigi Minuto, John D. Thompson, Gabriele Casazza

**Corresponding author:** Luigi Minuto, Department of DISTAV, University of Genoa, Corso Europa 26, 16132, Genoa, Italy luigi.minuto@unige.it

Table S1. Sampling design. Number of flowers (Flowers) used in different analyses and number of plants (Plants) on which they are. phen = measure of floral traits; Ps = pollen limitation; Po = open pollination; self = self pollination.

| Populations | Plants phen | Flowers phen | Plants Ps | Flowers Ps | Plants Po | Flowers Po | Plants self | Flowers self |
| --- | --- | --- | --- | --- | --- | --- | --- | --- |
| P01 | 18 | 23 | 3 | 3 | 9 | 11 | 14 | 20 |
| P02 | 32 | 33 | 11 | 13 | 21 | 26 | 16 | 20 |
| P03 | 11 | 16 | 5 | 5 | 11 | 12 |  | / |
| P04 | 8 | 10 | 4 | 4 | 7 | 8 |  | / |
| P05 | 8 | 10 | / | / | / | / | 8 | 10 |
| P06 | 23 | 27 | 7 | 9 | 18 | 22 | 11 | 15 |
| P07 | 12 | 20 | 2 | 2 | 5 | 6 | 14 | 17 |
| P08 | 11 | 18 | / | / | / | / |  | / |
| P09 | 34 | 37 | 8 | 9 | 30 | 37 | 10 | 15 |
| P10 | 26 | 31 | 11 | 12 | 25 | 32 | 12 | 17 |
| P11 | 23 | 25 | 1 | 1 | 2 | 2 |  | / |
| P12 | 31 | 31 | 7 | 8 | 18 | 22 | 11 | 15 |
| P13 | 18 | 23 | 10 | 13 | 15 | 19 |  | / |
| P14 | 7 | 13 | / | / | / | / |  | / |
| P15 | 26 | 36 | 11 | 14 | 20 | 24 |  | / |
| P16 | 15 | 20 | 3 | 3 | 4 | 4 | 12 | 15 |
| P17 | 25 | 31 | 4 | 4 | 33 | 42 | 13 | 15 |
| P18 | 34 | 37 | 22 | 28 | 24 | 30 |  | / |
| P19 | 30 | 80 | 22 | 27 | 20 | 28 | 10 | 12 |
| P20 | 22 | 41 | 12 | 13 | 17 | 19 | 8 | 12 |
|  | **414** | **562** | **143** | **168** | **279** | **344** | **139** | **183** |
